# Adenosine stress and rest T1-mapping differentiates ischemic, infarcted, remote and normal myocardium - a novel gadolinium-free method for ischemia detection with immediate applications in coronary artery disease

**DOI:** 10.1186/1532-429X-18-S1-O66

**Published:** 2016-01-27

**Authors:** Alexander Liu, Rohan S Wijesurendra, Jane M Francis, Matthew D Robson, Stefan Neubauer, Stefan K Piechnik, Vanessa M Ferreira

**Affiliations:** OCMR, University of Oxford, Oxford, UK

## Background

In stable coronary artery disease (CAD), accurate ischemia detection enables targeted revascularization, which improves clinical outcomes. Myocardial blood volume (MBV) may be a better ischemia marker than myocardial blood flow. Adenosine stress and rest T1-mapping is a novel gadolinium-free CMR technique that reflects changes in MBV through its water content. We hypothesized that different myocardial tissues in CAD patients and controls have distinctive T1-responses to adenosine, enabling ischemia detection without gadolinium agents.

## Methods

CAD patients (n = 13, 65 ± 10 yrs, >50% angiographic luminal stenosis in ≥1 coronary artery) and normal controls (n = 14, with n = 7: 28 ± 5 yrs and n = 7: 57 ± 13 yrs) underwent CMR at 1.5T to assess for LV function (cine), ischemia (adenosine stress/rest first-pass perfusion) and viability (late gadolinium enhancement, LGE). These were compared with novel adenosine stress/rest T1-mapping performed using ShMOLLI. T1 values of different myocardial tissues (infarcted, ischemic and remote in CAD patients; normal segments in controls) were estimated using manually placed regions of interest (ROI). ROI for infarcts were placed in the infarct core, carefully referenced to LGE and cine. ROI for ischemic myocardium were placed in regions corresponding to reversible perfusion defects on first-pass imaging, downstream significant angiographic coronary stenosis, and without evidence of LGE. ROI for remote myocardium were placed in areas free from perfusion defects, infarctions, upstream angiographic stenosis or wall motion defects.

## Results

**Figure 1:**
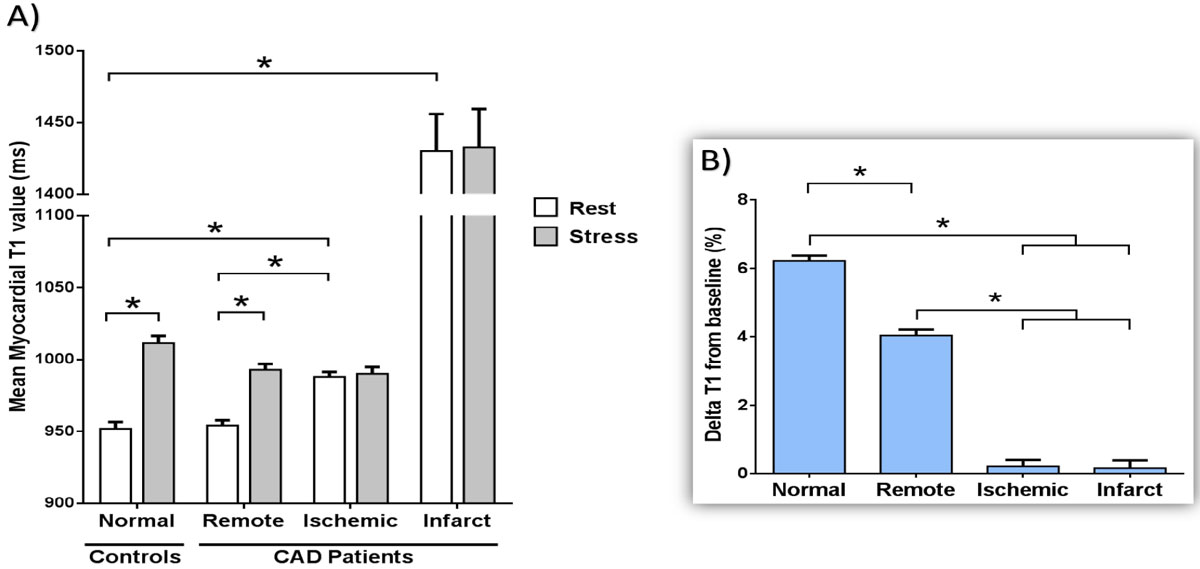
**Normal, remote, ischemic and infarcted myocardial tissues demonstrate characteristic T1-profiles (A) and stress T1-responses (B)**. All bars represent mean ± 1SEM. *denotes p < 0.001.

**Figure 2:**
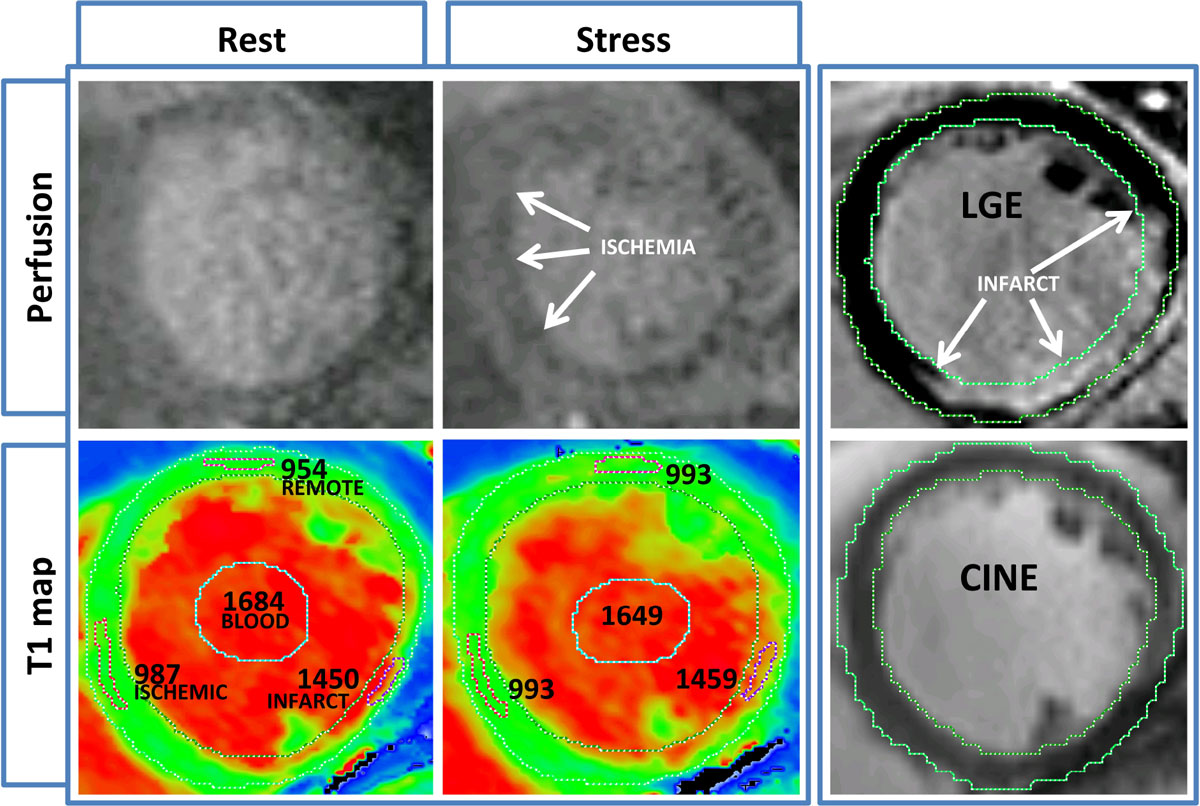
**T1-maps at rest and during adenosine stress of a patient with stable coronary artery disease, referenced to stress/rest perfusion, late gadolinium enhancement (LGE) and cine images**. Areas of interest are as labelled; all numbers represent mean T1 values (ms).

Normal controls showed normal resting myocardial T1 (952 ± 18 ms) and positive T1-reactivity of ~6% from baseline during adenosine stress (6.2 ± 0.5%, p < 0.001). Younger and older controls showed similar resting T1 (952 ± 22 ms vs 952 ± 13 ms, p = 0.95) and T1-reactivity (6.3 ± 0.6% vs 6.2 ± 0.6%, p = 0.77). In CAD patients, infarcted myocardium exhibited the highest resting T1 of all myocardial tissues (1430 ± 86 ms, p < 0.001), with no T1-reactivity (0.2 ± 0.8%). Ischemic myocardium displayed higher resting T1 than normal controls (988 ± 12 ms vs 952 ± 18 ms, p < 0.001), with no T1-reactivity (0.2 ± 0.7%). Remote myocardium of CAD patients (non-ischemic/non-infarcted) showed normal resting T1 (954 ± 13 ms), with blunted T1-reactivity (4.0 ± 0.6%, p < 0.001) compared to normal controls (6.2 ± 0.5%, p < 0.001, Fig. 1). Fig. 2 shows example T1-maps of a patient.

## Conclusions

We successfully developed and implemented a novel CMR method for ischemia detection in CAD patients based on T1-mapping and without the need for gadolinium. We showed for the first time that normal, ischemic, infarcted and remote myocardium can be differentiated by their characteristic stress/rest T1-profiles. Further investigation of the blunted T1-reactivity in remote (non-ischemic/non-infarcted) myocardium of CAD patients, compared to controls, may deepen our understanding of CAD pathophysiology. Stress/rest T1-mapping has the potential to become a powerful diagnostic and risk stratification tool for CAD.

